# Enhancing shared street accessibility in heritage sites for individuals with visual disabilities: a Canadian perspective

**DOI:** 10.3389/fresc.2024.1419446

**Published:** 2024-12-24

**Authors:** M. Lakoud, E. Morales, A. Ruiz-Rodrigo, I. Feillou, S. Mathieu, F. Routhier

**Affiliations:** ^1^ School of Rehabilitation Sciences, Université Laval, Quebec, QC, Canada; ^2^ Centre interdisciplinaire de recherche en réadaptation et intégration sociale (Cirris), Quebec, QC, Canada; ^3^ Département des Relations Industrielles, Université Laval, Quebec, QC, Canada; ^4^ Le ministère de la Culture et des Communications, Montréal, QC, Canada

**Keywords:** visual disability, shared street, accessibility, active transportation, public space, mobility, equity, active design

## Abstract

**Introduction:**

Heritage sites often pose significant accessibility challenges for individuals with visual disabilities due to their preserved architectural features and strict regulations against modifications. In shared streets, designed to encourage pedestrian use and reduce vehicle dominance, these challenges are exacerbated by the lack of tactile and directional cues for visually impaired users. This study, set in the context of Canadian heritage sites, explores how shared streets can be adapted to be more inclusive while respecting the integrity of historical environments.

**Objective:**

The objective of this study is to explore and propose practical solutions to enhance the accessibility of shared streets for individuals with visual disabilities within heritage sites, with a particular focus on preservation requirements.

**Methodology:**

The study adopts a three-phase methodology. First, co-design sessions were conducted with three groups of stakeholders: people with disabilities, caregivers, and experts in accessibility and heritage preservation. Second, a narrative literature review was undertaken to identify practices from existing research and urban planning cases. Finally, solutions were developed in collaboration with a design firm to create practical, adaptable prototypes that address the specific needs identified in earlier phases.

**Findings:**

The co-design sessions revealed several key challenges, including the lack of tactile references, difficulties with snow removal, and the need for clearer delineation between pedestrian and vehicular zones. Solutions such as tactile paving, modular bollards, and the use of contrasting materials were developed to address these issues. The narrative review reinforced the importance of universal design in heritage contexts and provided insights into successful implementations in other urban settings.

**Conclusion:**

The study concludes that shared streets can be made more accessible for individuals with visual disabilities by adopting a modular design approach that integrates tactile cues and adaptable urban furniture. These solutions ensure that accessibility and safety can coexist with heritage preservation, promoting inclusivity in public spaces. The research highlights the importance of stakeholder engagement in the design process and offers a replicable framework for improving accessibility in heritage sites globally. However, further field testing is needed to assess the feasibility and acceptance of these solutions within the regulatory constraints of heritage environments.

## 1 Introduction

Heritage contexts pose significant challenges for people with visual disabilities. This is largely due to uneven surfaces and high or narrow steps or pavements. Many of these sites are protected by strict laws, depending on the country, which prevent modifications that would improve accessibility. For example, in Quebec City, Canada, in order to make an intervention to make the physical environment more accessible authorization is required from the Ministère de la Culture et des Communications du Québec and certain responsibilities are delegated to the city. The process is lengthy and if the proposed modification is likely to have an aesthetic impact on the buildings surrounding and within the heritage context, there is a significant probability that the proposal will be rejected (1). This is where shared streets emerge as a promising solution to address accessibility issues, by reconfiguring the street layout to create more uniform surfaces and eliminating traditional sidewalks.

Shared streets, accessibility for people with visual disabilities and heritage sites are three elements that continue to have problems in blending well together. On one hand, we have the shared streets that were originally intended to give a greater importance to the pedestrian (2), which had been completely forgotten by the presence of the car. The idea was to create a space where all street users could coexist harmoniously, primarily by significantly reducing the speed to 30 km/h, since the default and common speed limit on local streets in Canada is 50 km/h (3). A significant amount of research has been carried out to assess the effectiveness of shared streets and their impact on the mobility, safety and quality of life of road users, since these types of streets are usually accessible for people with motor disabilities, since there are no physical barriers such as the sidewalk (4, 5). Reducing car dominance on shared streets promotes social interaction, increases physical activity for pedestrians and cyclists, and reduces the number and severity of road collisions (6).

Blind people on shared streets lack tactile and directional cues (7). Raised sidewalks, which often serve as a physical separation between different traffic zones, may be absent or less perceptible on shared streets. This can cause confusion, reduce confidence, and increase anxiety for pedestrians with visual disabilities (8). In addition, ground markings such as crosswalks or wayfinding strips may be insufficiently contrasting, making navigation difficult for them. In Nordic regions in particular, it is essential to carefully choose the material of tactile surfaces to ensure their durability. For example, although rubber may seem promising, it is not suitable due to its vulnerability to winter conditions (9).

Despite the accessibility success of shared streets for individuals with motor disabilities, and the existing research to improve the design practice of accessible streets (4, 5) many fail to explore some potential solutions to address inclusion for the visually disabled. Thus, the main objective of this manuscript is to deepen our understanding of the challenges and opportunities of shared streets, through people with disabilities perspective, specially people with visual disabilities and to explore possible venues of universal solutions, including the visually disabled. We intentionally chose to delve deeper into the experiences and perceptions of safety among individuals with visual impairments. This focus was due to the relative lack of attention and documentation in existing literature on how such individuals navigate and feel secure in these environments, compared to other types of disabilities

To address these challenges, a “universal design approach” was adopted in this study. This approach is further examined in subsequent stages, as outlined in the article titled *Preserving the Past, Embracing the Future: Co-Design Strategies for Achieving Harmony between Heritage Sites and Accessibility Needs*, which provides a more detailed analysis of the intersection between accessibility and heritage preservation, and explores co-design strategies as a means to achieve a balance between maintaining historical integrity and enhancing accessibility for all.

## 2 Methodology

To meet the objective of this article, the methodology is divided into 3 phases: Phase 1- Three co-design sessions with different key stakeholders were organized to identify the challenges and opportunities (10) of shared streets and explore possible solutions; Phase 2- A narrative literature review in databases and on the Internet was done, to find best practices regarding the points identified in the previous phase; phase 3- Developing solutions with the help of a design firm that was integrated to the team, based on what was found to be best practices.

### 2.1 Phase 1: co-design sessions

The three co-design sessions took place at Centre interdisciplinaire de recherche en réadaptation (Cirris; Québec, Canada) within 2 weeks between one another. The average duration of each session was 2 h, and each was organized for a different type of subject expert, (1) participants with different disabilities, (2) caregivers, and (3) experts in accessibility, heritage and architecture.

Participants for the first session were adults with disabilities responding to the following selection criteria: to live with a visible (physical, visual, normal aging process related) or invisible disability (autism, intellectual disability, hearing disability, chronic pain or fatigue); to be 18 or older; and to be able to communicate with the research team with or without aids or support. Snowball and convenience sampling was carried out. Several organizations related to the targeted disabilities (e.g., *Regroupement d’Organismes de Personnes Handicapées de la region 03*—ROP 03) participated to the recruitment process. Participants who had expressed an interest in taking part in the study and had previously been involved in another stage of the project were contacted by telephone to determine their eligibility, as this topic is part of a larger, more extensive project.

The second session aimed to gather caregivers of another person with any kind of disability, being adults and being able to communicate verbally. They were recruited through the participants of the previous steps of the project.

Finally, participants for the third session, included experts with a minimum of 5 years of experience working on heritage buildings.

The place that was chosen to contextualize the accessibility problems was the Champlain District at the Old Quebec in Quebec City, Canada (https://www.quebec-cite.com/en/old-quebec-city) and streets such as Saint-Joseph that includes some heritage buildings. The reason for choosing this heritage site was because it is one of the most important site in Canada and the research team is well familiar with the environment due to another project developed previously entitled *Experiencing accessibility of historical heritage places with individuals living with visible and invisible disabilities* (11).

The “starting point” for the discussion in the three sessions was Exhibition Road in London, UK (https://www.gardenvisit.com/gardens/exhibition_road_south_kensington), as an example of shared street. This street stands out for the fact that it is entirely on one level, offering a shared space for every type of road user (pedestrians, cyclists and vehicle drivers). Limiting vehicle speeds to 30 km/h helps reduce the risk of accidents, and promotes a soothing urban atmosphere (3).

Each session started by explaining the characteristics of the concept of the shared street, showing several pictures and examples found on the internet. Afterwards, participants were given the opportunity to express their views and the first author opened the floor to suggestions and ideas for improving accessibility, such as regular surfaces to circulate, among others. Since the needs and recommendations of blind people emerged relatively quickly in each session, the second author would start synthesizing the participants’ ideas from their descriptions and draw them in a flipchart in front of everyone, in order to validate the idea and, for the rest of the participants, to understand the idea and being able to enrich it. This process would continue until the idea took on a solid, well-founded form.

All sessions were recorded and structured according to a uniform outline. The comments and solutions mentioned in the first co-design session were taken into account to present them in the second session and so on in the third, in order to enrich and better define the solutions. The research project was presented on a PowerPoint presentation by the first author.

### 2.2 Phase 2

The elements and solutions identified during the co-design sessions were meticulously organized by the research team to delve deeper into best practices outlined in existing literature and online sources. This process paved the way for a comprehensive narrative review, which characterizes it as an overview of scientific literature on a specific topic, providing a synthesis of available knowledge (12). This method facilitated the integration of recommendations with established knowledge and incorporated received feedback. The selection of articles typically occurs without pre-established inclusion or exclusion criteria, with varying opinions on the use of criteria to assess evidence quality (13–16).

Concurrently, authors initiated the narrative review process by defining a comprehensive set of keywords encompassing various aspects of the topic. These keywords guided systematic searches on databases such as Geobase and Google Scholar, laying the foundation for a thorough exploration of relevant literature. The keywords used for the research included terms like “Visual disability,” “Disabilities,” “Shared street,” “Accessibility,” “Active transportation,” “Public space,” “Mobility,” “Equity,” “Active design,” “Woonerf,” “Pedestrian environment,” and “Orientation and mobility.” All searches were conducted in English.

As we delved deeper, we expanded our search to include grey literature^1^ sources, consulting Google and exploring a website called “collectivitesviables.org,” which offers a series of articles related to this theme. Specifically, we found a relevant case study titled “Montréal: Concilier rue partagée et accessibilité universelle,” available at collectivitesviables.org. This search also uncovered a series of publications discussing topics like slowing down speed and active transportation, particularly relevant given the similar winter conditions between Montreal and Quebec City.

### 2.3 Phase 3: developing solutions

An industrial design company named “Elabore” was hired, funded by the grant, to develop prototypes. This company was selected for two main reasons, the first one is that one of the owners of the firm is a wheelchair user and due to his lived experience, he has a sensitivity that we can hardly find elsewhere, and the second reason is that one of the employees was involved in the project when he was a student in its initial phase, so the knowledge and awareness of accessibility issues, we found indispensable for the successful development of the project.

After signing a contract, a meeting of at least 1 h was held every week from October 2023 to March 2024. In these sessions, the company's progress was presented, and discussions were held to improve the proposals. In the case of a particular prototype, a full-scale wooden model was built to identify problems and find solutions. This particular session took place on the company premises with both teams, the research team for this article and the company team. After this, some weeks later, the prototypes were built and delivered to the research team.

## 3 Data analysis

Every co-design session was integrally transcribed by one team member, and the transcripts were revised by the first author (ML). A simple thematic analyses were conducted by associating the different solutions that were commented and commented again in each session. This type of analysis is recommended for exploratory studies where data is limited (17, 18). A similar approach was taken for the narrative review, meaning that the most important themes that emerged from the different sources were identified and regrouped.

These solutions from the co-design sessions were then compared to the results found in the narrative review.

### 3.1 Ethics

The study was approved by the sectorial ethics committee on research in rehabilitation and social integration of the *Centre Intégré Universitaire de Santé et de Services Sociaux de la Capitale-Nationale* (#2022–2422) and every participant signed a consent form.

## 4 Results

### 4.1 Co-design sessions

#### 4.1.1 First session

Seven participants (*n* = 7) were involved and presented different disabilities 2 participants with physical disabilities (P1 and P2), 1 person with visual disability P3, 1 deaf person P4, 1 person with autism spectrum disorder P5, 1 person with intellectual disability P6, and 1 elderly person P7, allowing for a variety of perspectives.

The proposal for shared streets, using London's Exhibition Road as an example, sparked lively debate. The idea offered a promising vision for meeting the needs of people with disabilities, while improving the usability of urban space for all citizens. However, it was noted that specific adjustments would need to be made to fit to the Champlain's District at the Old Quebec context. For example, a participant P1 in a wheelchair asked: “*Could ice be forming on the drain, complicating the situation?*” which extended the debate to the possibility of heating the drain, which would be beneficial in preventing snow accumulation in winter, but this option comes with significant costs.

Feedback from participants was essential to clarify that the shared streets solution did not include a recess for the drain, but rather a smooth surface. In addition, the use of sandstone with a fine joint for the cladding was emphasized to minimize shaking during movement. A pertinent question was posed by a participant P3 with visual disability: “*If it's flat, how does a blind person get around safely?*”

Concerns were raised by P3 about the practicality of snow removal, with the participant noting, “*It's good for snow removal, but there's a concern about the drains that could block the wheelchair wheels.*” This observation underscored the importance of a smooth surface, on the same point, P1 added: “*Well, for me, it was just…  I think it's really great [laughs], but I know one thing, it's that since these are stones, we need to make sure that the distances… you know, that there aren't any cracks between each stone because in a wheelchair, it's bump after bump after bump, and it's bad for… well, it hurts me a lot. I know that most people also have back pain because of that*”, leading to the decision to include raised tactile tiles strips on streets to guide the person with visual disability safely and to implement bollards to provide a sense of security. Additionally, the potential for climate change to reduce the costs of such implementations was discussed, as P3 remarked, “*With climate change, it's going to become less and less expensive…*”

If we can summarize the most important results of the first session, two main themes that emerged from the first session were winter and snow removal, as well as the need for more tactile references for blind individuals. It is important to bare in mid the context in which this research was developed. Quebec City receives more than 3 m of snow every winter, so it is not surprising that one of the biggest concerns for people with disabilities is precisely the snow.

#### 4.1.2 Second session

The second co-design session, 4 family caregivers participated, and special attention was given to their contribution as they provided an additional valuable perspective. As daily supporters of people with disabilities, they offer a unique insight into real and practical needs. This helps develop more suitable solutions by considering both the expectations of the individuals being assisted and the challenges faced by their caregivers.

The two main issues that came up in the previous session (snow removal & tactile references) were addressed by the research team and a proposal of solution was included in the presentation.

**Figure 1:**
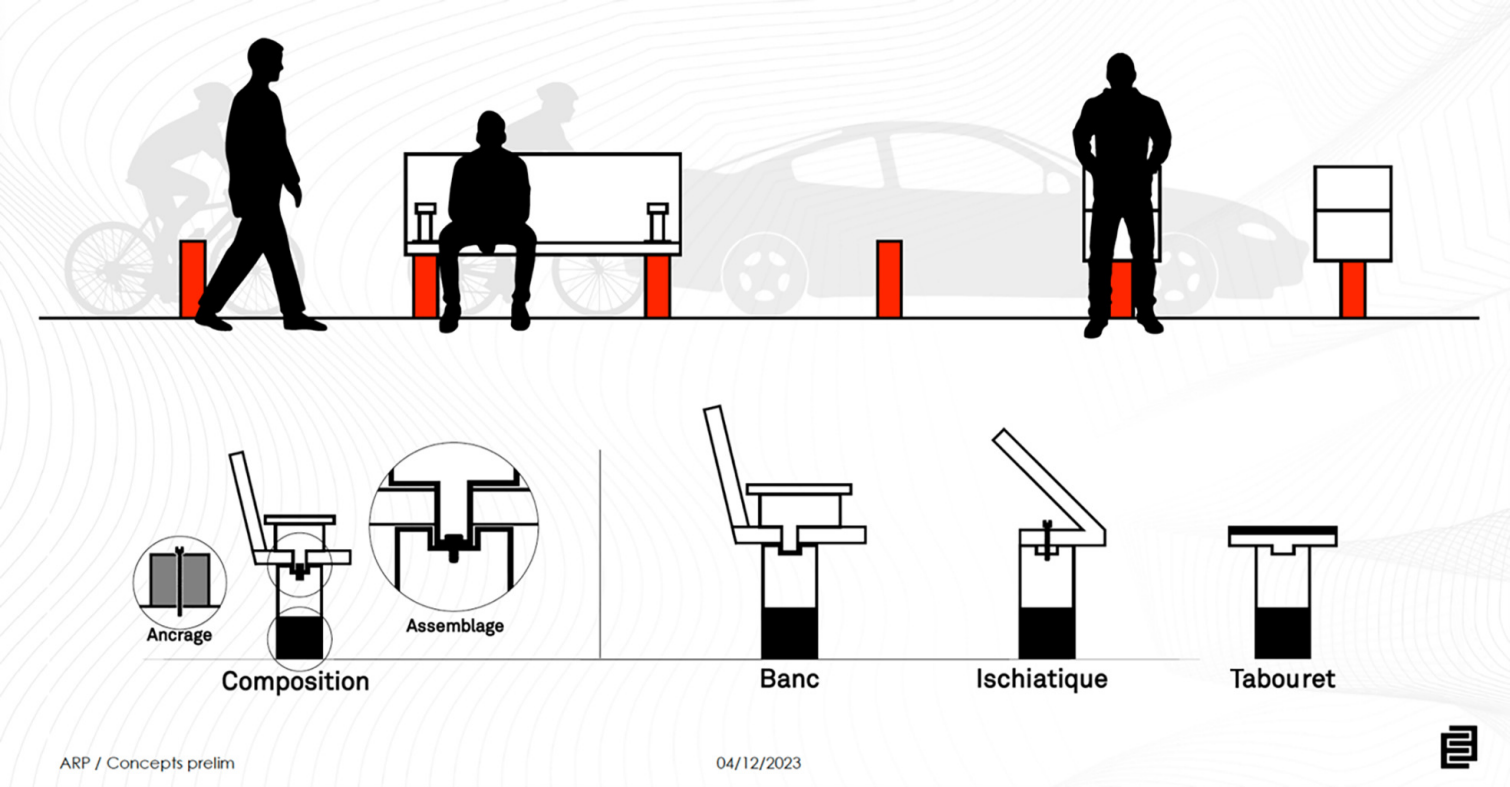
Elevation section explaining the Lego concept.

**Figure 2:**
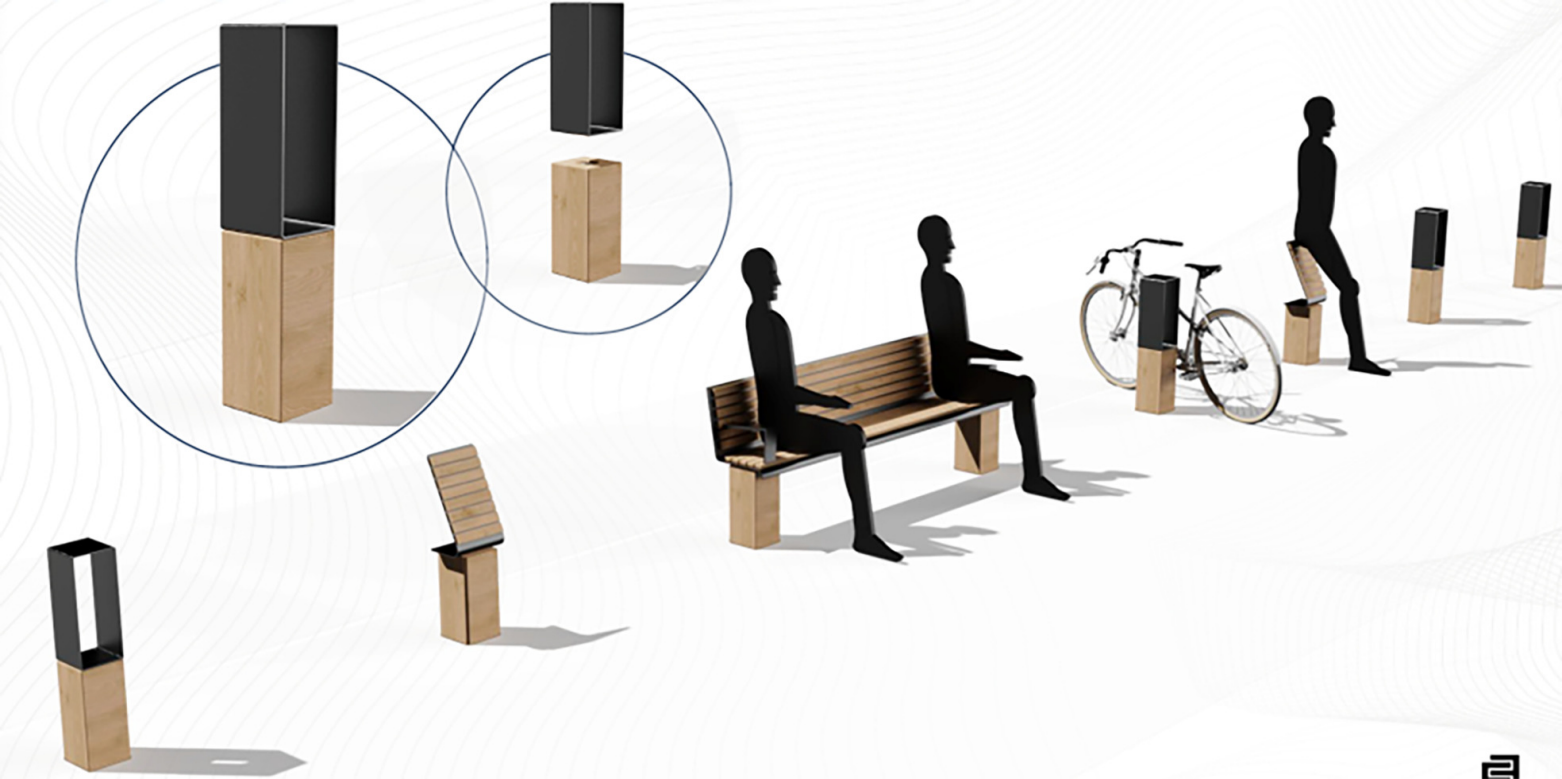
Oblique view of the concept.

A caregiver of a person in a wheelchair (P1) highlighted the challenge faced by wheelchair users in such weather conditions, emphasizing the need for a solution like vertical wrist handles to ease maneuverability. The observation was that the addition of a handrail could considerably ease the passage of wheelchair users by providing extra support, as shown in the figure below, P1**:** “*For the ramps, if we could also add some handles because usually, it's like full of snow and the bars… It's hard to grab with your hand. A vertical handle could help because it's for someone in a wheelchair*”. However, the delicate issue of winter and snow quickly emerged. It was pointed out that the handrails were likely to be covered in snow, making them difficult to use. After some debate, an emerging solution was proposed: integrate vertical supports on the handrails, thus limiting snow accumulation (Figures 1, 2). This idea would maintain the handrails’ functionality throughout the winter, ensuring easier and safer access for people with reduced mobility in Old Quebec. This idea underlining the need to think about practical, ergonomic devices to ensure unhindered mobility.

The thorny issue of winter snow management was addressed, with the proposal of an innovative solution inspired by Iceland, which exploits geothermal energy to heat certain sections of sidewalk. A proposal was made to incorporate a transparent coating for ground leveling without obscuring the tiles. “*For sidewalks with floor tiles, a substance can be added to flatten the floor without masking it*” said P1 creating a link with previous considerations on street surfaces.

P2 who is a second caregiver of another person in a wheelchair said: “*We can also work with… I would say paving stone, anti-slip stone but with a joint that is like 1 mm so that it doesn't stir*.” By using anti-slip stones with minimal joints, they aimed to mitigate the risk of displacement or tripping hazards, ensuring a smoother and safer walking surface. However, this solution was not popular, because the concern was about removing everything and replacing it with new sandstone, which would be expensive.

To enhance inclusivity, some participants suggested engraving grooves in the pavement to serve as tactile strips, guiding the walking sticks of blind individuals and making navigation easier.

The accessibility of bus stations was also discussed, using the example of the Place d'Youville^2^ station as an illustration of the importance of making all stations accessible, especially for wheelchair users. P1: “*Okay. the most important thing, is to make all the bus stations accessible because …. my friend, sometimes he doesn't go out into the city just because, for example… the d'Youville station isn't accessible. So it's a lot of walking to get down to these places.*”

The potential benefits of heated sidewalks were highlighted, alongside the idea of signage on solar-heated metal surfaces, suggesting an innovative approach to making urban spaces more comfortable in all seasons. However, it's not a proposal feasible in the Quebec context, as a northern country where snow takes up a lot of space and heating with electricity would be very expensive and in Quebec there are no alternative options such as geotherm or solar panels as there is not that much sun in wintertime.

The same themes from the first session were discussed again in the second session (winter, providing better references for blind people on the street), this time with a wider range of potential solutions.

#### 4.1.3 Third session

During our third co-design session, a sample of *n* = 7 experts with diversified skills, from different disciplinary spheres. These experts, came together to assess the viability and relevance of the ideas co-created in the previous phase.

The experts selected came from different backgrounds such as architecture, heritage and accessibility. Their practical experience of working with people living with disabilities was also a came as a valued asset. What's more, some of the experts were direct representatives of the sites concerned, bringing a relevant and concrete perspective to the discussions from the point of view of workers, managers and owners.

P1, who is an occupational therapist, expressed her initial impression regarding shared streets, stating, “*Blind individuals often experience a lot of discomfort on shared streets.*” This perspective highlights the challenges faced by visually impaired people in navigating urban environments that do not adequately accommodate their needs, emphasizing the importance of designing inclusive spaces that prioritize accessibility and comfort for all users.

Some participants (P2 and P5) who are respectively a professor of architecture at the faculty of architecture at Laval university and an architect raised the idea of removing sidewalks to bring heritage sites closer to the past, referring to the fact that at a time when horses reigned supreme, there were no sidewalks. They also suggested that this approach would be easy and gradual, stressing that it was a simple solution, if not a strategy. However, concerns were expressed about snow clearance on streets without sidewalks by a disability advisor at Laval University (P3), and the potential problems associated with the restaurant and bar terraces that currently occupy these spaces was raised by the representative of the merchants of Petit Champlain Street (P6).

In addition, the issue of cyclists was raised by an occupational therapist (P1), with the idea that they can be perceived as an additional challenge on shared streets, and proposals to introduce guides or maps to orient people were made. The session also highlighted specific situations where compromises have to be found, notably about emergency situations (P5) and the need for crossings to reach sidewalks on one side (P1). The issue of changing the street level was raised, warning of the potential impact on building structure (P2).

The perspective of blind people was put forward (P1), pointing out that tactile cues are not sufficient and that eliminating sidewalks would add a significant burden for blind people, causing a sense of insecurity. It was suggested that adding gradients (P3) might be a more appropriate solution, and the idea of projecting routes in winter to increase contrast on the street was mentioned (IF). Some participants noted that familiarization with this solution, combined with appropriate signage, could facilitate its acceptance. Some new ideas came up, for example, to put 2 podotactile strips, instead of one, to show the boundary of the pedestrian zone. One at the side of the buildings and the other at the boundary for cyclists or motorists. In the same way it was suggested to use street furniture to increase the references for blind people.

With regard to snow, it was suggested that some street furniture could be easily removed in winter to facilitate the task of snow removal.

### 4.2 Narrative review

A total of 110 sources, including articles and reports, were consulted. This review encapsulated comprehensive insights into various aspects, highlighting key findings in the areas of pedestrian and cycling delineation, surface coating, signage and lighting, and urban furniture. These topics provided a comprehensive understanding of the considerations and recommendations associated with creating accessible and well-designed environments. The diverse range of sources accessed through Google Scholar and Geobase enabled us to construct a framework for addressing the complexities of urban planning and design in relation to accessibility and mobility.

We selected articles dating from 2000 to 2023 to gain insight into discoveries made over the past decades, with the most recent research retained dating back to 2005. The following paragraphs describe the most recurring themes mentioned in the various sources of the review, along with those that resonated most with the results of the co-design sessions.

#### 4.2.1 Delineation of pedestrian and cycling

A recognizable structure that clearly indicate places designated for walking and crossings were identified as essentials. This recognition relies on the coherence and predictability of the environment, characterized by elements with specific meaning and function. Thus, each potential destination should be accessible through a recognizable, obstacle-free, and uninterrupted path, ensuring navigation without confusion (19). To ensure effective separation between the pedestrian zone and the roadway, as well as the cycling lane, protective strips with a minimum width of 60 cm can be utilized. These strips should be tactile, allowing identification using a detection cane or foot, for example, using a green strip (9).

#### 4.2.2 Surface coating

The material for tactile surfaces must be thoughtfully chosen, especially in Nordic countries, to ensure good durability. The coating material is non-slip under all weather conditions, does not produce reflections, and minimizes glare. There should be no space between the pavers or slabs (20). To this end, the coating must not have holes, gaps, or other deformations, and there should be no steps exceeding 13 mm (Advisory Committee on Universal Accessibility, 2014). A British report emphasizes the importance of surface drainage to prevent water accumulation. It recommends the installation of linear drains along tactile delineators (21). The careful consideration of these coating specifications ensures not only the longevity of the material but also the safety and accessibility of the environment, particularly in adverse weather conditions.

#### 4.2.3 Signage and lighting

The illumination should be sufficient, activated as needed, arranged in a thoughtful and consistent manner, and avoid any light pollution. For instance, employing a combination of streetlamps and urban furniture can be utilized to physically mark the separation between pedestrian and vehicular areas (19). Signage should be easily visible for individuals with residual vision and should be designed with high contrast and large characters (22). The entrance and exit of a shared street must be clearly recognizable and announced in a straightforward manner (23). The installation of two-dimensional tactile maps (with raised points adhering to specific standards) or three-dimensional maps (in the form of models) can greatly enhance the understanding of an unfamiliar environment by facilitating mental representation (24). Bollards or poles could serve as carriers of tactile navigation assistance information, particularly to indicate the direction and relative distance to the nearest crossing point (25).

#### 4.2.4 An imaginary tunnel

When setting up a pedestrian corridor marked by street furniture, it's important to follow certain guidelines to optimize accessibility. According to the study titled *Clinical Programming 2022 Institut Nazareth et Louis Braille: Final Report* (26), furniture components should not be separated by more than 1.2 m, thus promoting a coherent, unobstructed path. In addition, the lighting of these elements proves essential to enable visual discrimination for people with visual residuals, in line with the recommendations of Parkin and Smithies (27). As far as planting areas are concerned, they require particular attention to avoid encroaching on the pedestrian corridor. As stated in the study titled *Clinical Programming 2022 Institut Nazareth et Louis Braille: Final Report* (26) they should have a minimum width of 1.2 m where trees are present, or 0.6 m in other cases. In addition, the planting pit must be level with the pedestrian corridor, with a maximum difference of 6 mm, and plantings must be regularly maintained.

#### 4.2.5 Urban furniture

Street furniture components must be designed to be detectable with the cane, following the concept of the “imaginary tunnel” evoked by some authors such as Isler, Dejeammes and Hallet (28). Thus, careful planning is required to define a sufficient volume to allow an obstacle-free path for the visually disabled. Urban furniture encompasses various elements such as fountain posts, parking meters, benches, bus stops, poles, bike racks, waste bins, and planting zones. When the pedestrian corridor is marked by urban furniture, the components of the furniture should not be separated by a distance of more than 1.2 m (29). Additionally, they should be illuminated to allow discrimination by individuals with residual vision (27). Every component of equipment or urban furniture must be detectable with a cane. Some authors refer to the concept of an “imaginary tunnel” originating from Barcelona to keep in mind the necessary volume delineating an obstacle-free path (28). Planting zones should not encroach on the pedestrian corridor. Hence, they must have a minimum width of 1.2 m when including trees or 0.6 m in other cases. Additionally, the planting pit should be at the same level as the pedestrian corridor or with a maximum difference of 6 mm, and the plantations require regular maintenance (29).

#### 4.2.6 Maintenance and seasonal conditions

A Quebec study involving 24 individuals with visual disabilities and 12 orientation and mobility specialists found that warning tiles made of polymer (Armor-tile) or stainless steel (Advantage) remained detectable underfoot in winter conditions in sunny areas. Armor-tile achieved the highest detectability score. However, the authors note certain nuances: the impact of color on detectability could not be satisfactorily analyzed, and the study in semi-controlled conditions would benefit from being complemented by a study in real conditions where the tiles would undergo regular maintenance such as snow removal and abrasive spreading (30, 31).

### 4.3 Developing prototypes

With the different results found in the co-design sessions and the narrative review, it was decided to try to create a proposal including as many of the results as possible. However, the most important emphasis was put on the creation of shared street elements to promote a feeling of safety for visually disabled people. Therefore, our proposal revolves around the design of a shared street that do not share areas and where the three lanes—bicycle, pedestrian and car—cohabit at the same level (no difference between sidewalk and road) but are clearly identified. In order to address the specific concerns of the visually disabled, we suggest the integration of removable bollards. These will play a crucial role as a physical barrier, while scrupulously respecting White's requirements, notably by avoiding the use of materials that generate a dazzling effect (20).

As part of our collaborative teamwork, we set about synthesizing our ideas, taking into account the constraints inherent in the urban context, as well as the various needs of users, with particular emphasis on the challenges posed by harsh winters. The solution that emerged from this process is a “modular concept” or “LEGO-like concept” applied to urban planning in cities like Quebec City. This concept features a range of components (pedestrian benches, bollards, signage, barriers to slow down cars and bicycles) that allow for flexibility and customization, enabling the heritage environment to be shaped and adapted harmoniously to local specificities.

## 5 Discussion

This article aimed to deepen our understanding of the challenges and opportunities of shared streets, through the perspective of PWD and to explore possible venues of universal solutions that fulfilled the needs of visually disabled individuals. The results from the co-design sessions and the narrative review align in recognizing the challenge of blind or visually disabled pedestrians circulating in shared streets. Navigating in an unfamiliar or atypical environment increases the cognitive load for any pedestrian. While sighted pedestrians can compensate for this load with visual cues at their disposal, visually disabled pedestrians may optimize the auditory and perceptual information available (32). This unf is more palpable in environments where design features are not uniformly comprehensible to these individuals. Obstacles, such as the absence of visual cues, clear demarcation between spaces intended for cyclists and pedestrians, insufficient signage at street corners and crossings, and the non-existence of dedicated crossing zones, were identified (33, 34), promoting a feeling of insecurity. However, the results of both, narrative review and co-design sessions, shed light in contributing to pedestrians’ comfort by reducing traffic volume and speed (35). Nevertheless, the claim that the reduction of demarcations alone decreases traffic speed lacks empirical support and requires further discussion.

In most cases, the average vehicle speed diminishes in shared streets with higher pedestrian density, aligning with the goals of such zones. Nevertheless, it's crucial to consider factors such as eye contact, as highlighted in the study by Karndacharuk, Wilson, and Dunn (36), raising awareness that auditory information from traffic might diminish in these configurations. Additionally, the advent of quieter electric or hybrid vehicles presents a new challenge for visually disabled individuals (Canadian Council of Physical Transport Administrators, 2013; 32). Thus, the discussion on shared streets should encompass the multifaceted aspects of traffic reduction and the evolving landscape of vehicle technology.

Another aspect that converged in the narrative review and the co-design sessions was incorporating differences in color, materials, or a slight gradient in crossing zones that can effectively encourage motorists and cyclists to reduce speed and exercise greater caution. Additionally, strategically narrowing the road at specific spots can naturally induce a reduction in speed, aligning with the recommendations of Havik and Melis-Dankers (19).

There were some elements that the co-design sessions did not covered and the narrative review complemented and helped to have a better understanding. For example, additional elements that need to be taken into consideration such as having an adequate path that remains intrinsically conditioned by its continuity, the visibility inherent in its routes, its safety and comfort, while taking into account accessibility-related aspects (37). According to the recommendations of the Comité consultatif en accessibilité universelle (38) from the Provincial Government of Quebec, a pedestrian corridor should be characterized by its straightness and continuity, avoiding any potential obstacle forcing the pedestrian to deviate from his or her trajectory. Yet even with such a path, the indicators for entering and exiting a meeting zone frequently remain insufficient, as Havik et al. (16) and Melis-Dankers et al. (39) have pointed out. In particular, a clear tactile demarcation between sidewalk and sidewalk is a fundamental requirement.

On the other hand, the co-design sessions shed light into the fact that if we opt for the installation of specific lanes and paths dedicated to each type of road user, there is still a risk that cars will dominate the space and not feel obliged to slow down or share the road fairly. This approach could compromise the safety of other road users, such as pedestrians, cyclists and people with reduced mobility, who could find themselves in conflict with vehicles. Moreover, implementing a shared street in a Nordic City may increase some safety issues. There are multiple concerns: drivers’ adaptation to an approach devoid of visual cues, the risk of vehicles dominating specific lanes, and the potential conflict with the aesthetics and integrity of heritage sites. Rigorous analysis of these considerations is imperative to ensure the safety, accessibility and preservation of sites and road users.

By adopting the shared street strategy, we aim to create an environment where pedestrians have a decisive influence on traffic flow. However, for this approach to be effective and following the line of thought of the participants in session 1, it's essential that the crossing zone is clearly identifiable and discernible, thanks to visual and tactile cues. A precise direction must be indicated, enabling visually disabled people to easily locate the beginning and end of the crossing zone, as well as the guidance line indicating the path to follow. These tangible markers are an ideal replacement for the perpendicular kerb that visually disabled people generally use to cross in a straight line. This approach, advocated by Havik and Melis-Dankers (19), aims to create a safer and more intuitive crossing experience for visually disabled pedestrians.

After in-depth discussions and careful analysis of the data collected, and despite the warnings in the narrative review highlighting the potential risks associated with bollards for the visually disabled, our proposal is based on an approach carefully adapted to the Quebec context, particularly the heritage and winter ones. Mindful of White's recommendations that bollards are often considered the most dangerous elements of street furniture for visually disabled individuals, we advocate an innovative solution (20).

The modular approach enables urban design to be conceived as a creative and evolutionary process. This methodology offers an accessible and adaptable solution for heritage environments, particularly for northern cities like the Old Quebec City. By envisioning the urban site as a green base, the pieces become a metaphor for street furniture and landscaping. As in the construction of a modular structure, these elements can be added, shaped and installed to optimize the opportunities offered by the site. This approach promises a grounded approach to to respond to the complex requirements of urban design, integrating climatic imperatives and the varied needs of users.

It should be stressed that this approach goes beyond simply separating traffic flows. We see these bollards as modular elements, reminiscent of the “LEGO-like concept” in which you can interchange “pieces” in order to better adapt to the physical context of the street responding at the same time with pedestrians (visually disabled or not). This flexibility would enable the urban space to be dynamically adjusted to the changing needs of the community. Referring to the work of White, who advocates a minimum height of 1 m for bollards, as well as adequate visual contrast, our proposal aims to integrate these elements into a versatile system, serving as a base for additions such as benches, buttock rests, bicycle parking facilities, and other functionalities, to create an inclusive and evolving urban environment.

These bollards would act as physical barriers between the different lanes, providing clear and tangible markers for pedestrians, cyclists, and motorists. The introduction of these bollards aims to create an environment where each road user has a clearly delineated space, helping to reduce the risk of conflicts and accidents, providing, at the same time, a feeling of security for the visually disabled.

One of the key features of these bollards is their removability. This design has been carefully thought out to meet the specific climatic requirements of the Quebec context, where snow and winter conditions play a major role. Removable bollards enable snow removal crews and plows to carry out their operations throughout the winter, without impeding traffic flow or compromising safety. This flexibility is crucial for ensuring the year-round practicability of shared streets, accounting for seasonal variations and the weather challenges unique to the region.

By incorporating removable bollards and other urban furniture in shared streets, both the needs of the visually disabled and the practical imperatives of managing public spaces in a demanding winter environment will be taken into account. This measure aims to balance safety, accessibility, and functionality, while ensuring a safe and pleasant experience for all road users, whatever the environmental challenges facing the Quebec region.

### 5.1 Example of the application of our findings: making Rue Saint-Joseph inclusive

Located in the heart of the Saint-Roch district in Québec, Saint-Joseph Street is a vibrant hub of activity, close to the downtown area. It seamlessly blends the old with the new through its boutiques, restaurants, and cultural spaces, showcasing a successful urban revitalization. Its strategic location makes it easy to explore the various facets of the city, making it a favored spot for both locals and visitors alike. Saint-Joseph Street is synonymous with dynamism and culture, reflecting the lively spirit of Québec. It hosts a variety of shops, restaurants, bars, and cultural venues, offering an interesting mix of old and new. The street plays a significant role in the cultural life of Québec, hosting various events and festivals throughout the year.

We chose Rue Saint-Joseph as a prototype street for its potential to be not only accessible but inclusive for people of all abilities. With its width of 12,000 mm, the street offers ample space to accommodate various types of users, including pedestrians, cyclists, and motorists. This width allows for the implementation of features and designs that prioritize inclusivity, such as widened sidewalks, designated bike lanes, and traffic-calming measures.

**Figure 3:**
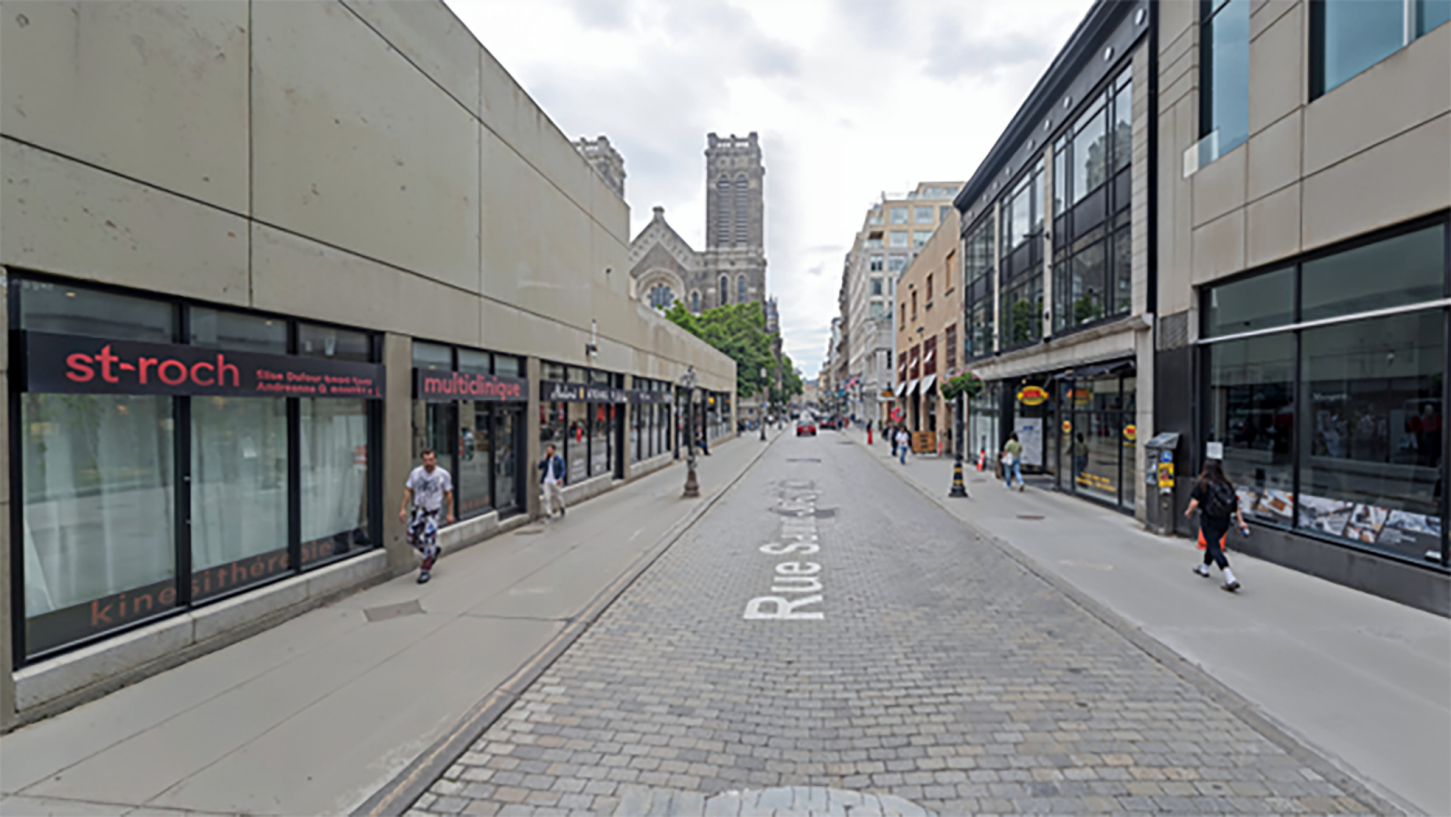
“Rue Saint-Joseph”: the heart of Saint-Roch, Québec.

Figure 3 shows the current state of Saint-Joseph Street. The image depicts a street with sidewalks on both the right and left sides, with a noticeable absence of clear demarcation or a safe zone for pedestrians. This lack of defined pedestrian areas highlights the challenges faced in ensuring accessibility and safety for all users.

**Figure 5:**
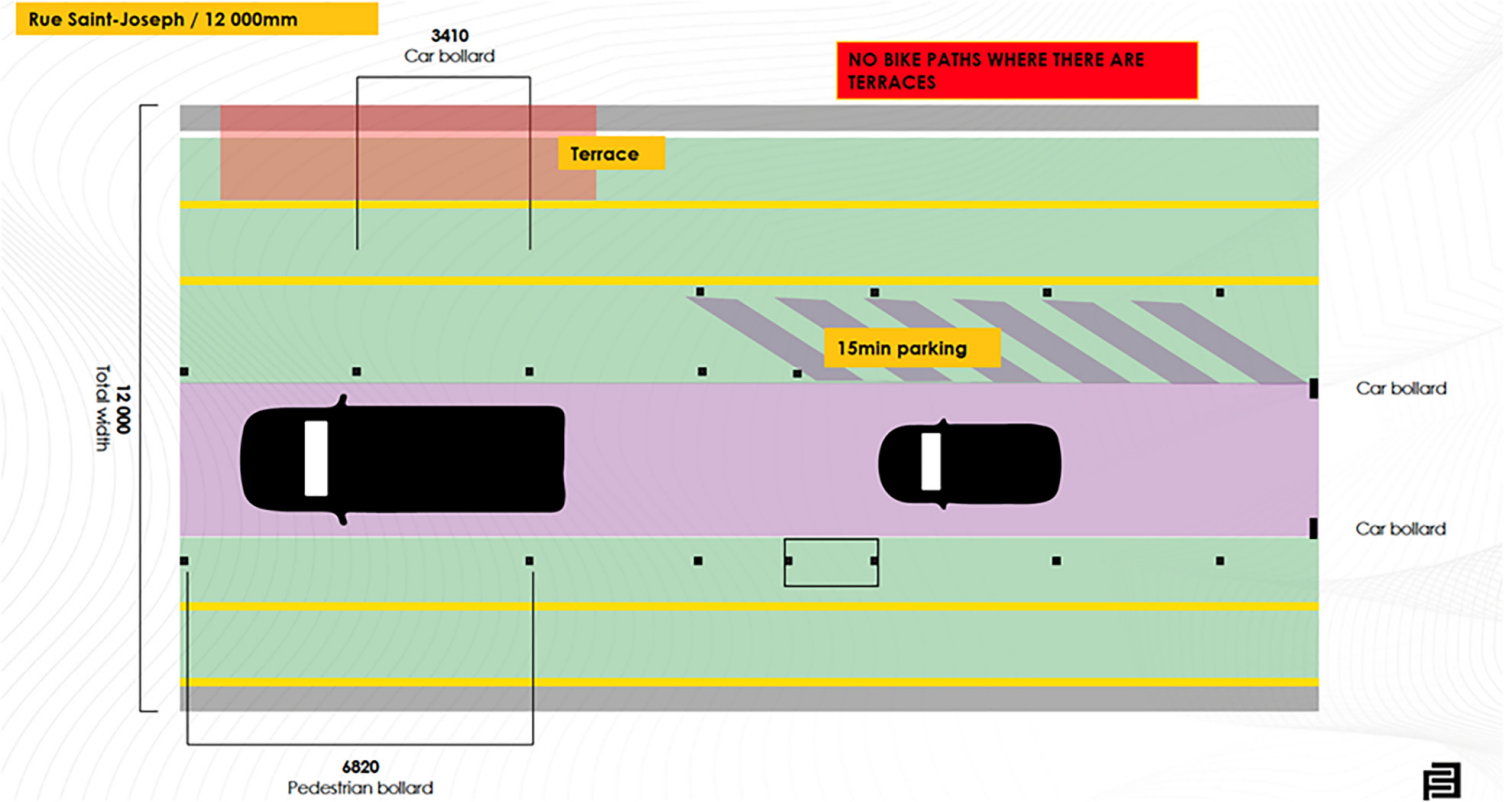
Prototype design for the Rue Saint-Joseph.

By selecting Rue Saint-Joseph, we aim to demonstrate how urban spaces can be redesigned to ensure accessibility and inclusivity for everyone, regardless of their mode of transportation or physical abilities.

**Figure 4:**
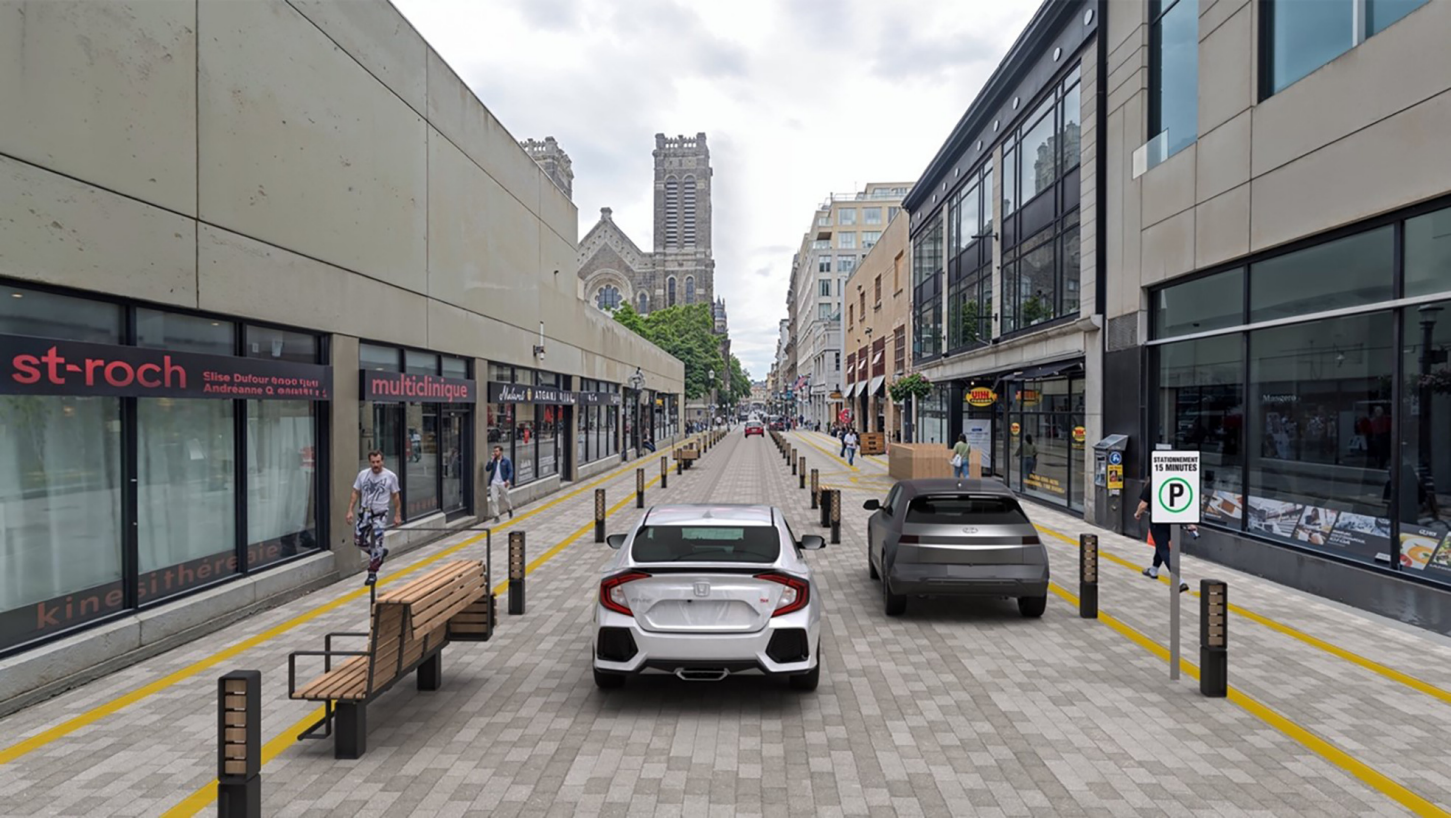
A design proposal for “Rue Saint Joseph”.

The two-dimensional proposal illustrated in Figure 4, and the three-dimensional proposal shown in Figure 5, present a more structured street layout with a clearly defined pedestrian zone, featuring bollards to enhance the sense of safety for all types of users within the pedestrian area, as mentioned by the participants of session 1. Tactile paving is present to guide individuals with visual impairments, and the removal of sidewalks aims to offer a more accessible environment for those with mobility impairments. Additionally, a designated parking area is planned to improve accessibility for motorists, making the street more user-friendly for everyone.

However, we had to consider the seasonal installation of outdoor terraces, particularly during the summer months. This installation significantly alters the level of activity, the associated risks, and the flow of traffic on the street. As a result, we made the decision to temporarily exclude the bike lane from this street during these periods. This measure was taken to mitigate the potential increased danger that the bike lane would pose to all users, including restaurant patrons, servers, pedestrians, and even cyclists themselves.

An important limitation of the present study is to test the proposal. We do not know if this proposal will be accepted by the Ministère de la Culture et des Communications du Québec, as it will certainly have an aesthetic impact on the heritage context. However, we wanted to integrate noble materials such as wood and steel for a better integration of the proposal in the context. However, studies such as the present one, may contribute to give guidelines to integrate these solutions in the future.

## 6 Conclusion

The design of shared streets represents a complex and multifaceted challenge, requiring a holistic approach that integrates the needs of blind and visually disabled pedestrians, road safety requirements and the aesthetic and functional constraints specific to each environment. The concept of “the exhibition road”, although successful in London, raises questions as to its transposition to a Quebec context, where the specificities of road behavior and infrastructure diverge. It is imperative to reconcile accessibility requirements for the visually disabled with safety requirements for all road users. The adoption of clear, consistent visual and tactile cues, the provision of continuous, obstacle-free footpaths, and the integration of solutions adapted to local particularities are all prerequisites for ensuring harmonious, safe coexistence.

Implementing a shared street, for example, in the heart of the Petit Champlain district in the Old Quebec or at Saint-Joseph Street, presents considerable practical challenges. Testing such a configuration with people with disabilities raises major concerns in terms of actual accessibility. Shared streets require particular attention to safety and ease of use, especially for people with disabilities. Therefore, while prototypes can offer useful insights, the actual integration of these concepts into real environments requires thorough evaluation and constant adaptation to ensure an inclusive and safe experience for all citizens, regardless of their abilities. However, this model aspires to be an inspiring source for future initiatives, providing a foundation for projects that seek to reconcile universal accessibility with heritage preservation. This vision encourages collaboration among researchers, practitioners, and decision-makers, thereby creating a conducive platform for the emergence of avant-garde solutions in the realm of inclusive urban planning that respects cultural heritage.

Ultimately, striking a balance between accessibility, safety and aesthetics in shared streets within a heritage context remains an ambitious goal. However, it is by adopting an inclusive approach, drawing on international best practices while adapting them intelligently to local realities, that we can hope to create urban spaces where the mobility and autonomy of blind or visually disabled people are fully considered, while promoting the fluid and respectful cohabitation of all road users.

The study's main contributions include providing a deeper understanding of the challenges faced by people with visual disabilities in shared street environments, particularly within heritage contexts in Canada. It also explores potential universal design solutions that could enhance accessibility while preserving the cultural and historical integrity of such sites. The research highlights the importance of co-design with experts from various fields, offering a collaborative approach to solving accessibility issues. Furthermore, it brings to light the specific needs of visually impaired individuals in urban planning, contributing to the broader discourse on inclusive design.

However, the study also has limitations. These include a potentially narrow focus on Canadian contexts, which might limit the applicability of the findings in other regions with different cultural, legal, and environmental conditions. The reliance on a relatively small number of experts (*n* = 7) for the validation phase might also restrict the diversity of perspectives included in the analysis. Additionally, the study's conclusions are drawn from a combination of literature review and expert opinion, which may not fully capture the lived experiences of people with visual disabilities. Specifically, only one person with visual impairment and one caregiver of a visually impaired person were consulted, which may not adequately represent the broader spectrum of experiences and needs within the visually disabled community. Future research could benefit from more extensive field studies and direct engagement with a larger and more diverse group of individuals with visual disabilities to validate and expand upon these findings.

## Data Availability

The raw data supporting the conclusions of this article will be made available by the authors, without undue reservation.

## Footnotes

^1^**Grey literature**: refers to materials and research produced by organizations, governments, academia, or industry that are not formally published through traditional commercial or academic publishing channels. These documents are often not subjected to rigorous peer review, which is a key distinction from more formal academic publications.

^2^Place d'Youville in Quebec City is a square located in Old Quebec. It's a lively place with cultural activities and restaurants, and is surrounded by the historical atmosphere of Old Quebec.

It presents accessibility challenges due to its elevated location, steep slopes and cobblestone streets. These features can make access difficult for people with disabilities, requiring special attention to access and travel planning.
