# Erratum: Enhancing shared street accessibility in heritage sites for individuals with visual disabilities: a Canadian perspective

**DOI:** 10.3389/fresc.2025.1629020

**Published:** 2025-05-30

**Authors:** Frontiers Production Office

**Affiliations:** Frontiers Media SA, Lausanne, Switzerland

**Keywords:** visual disability, shared street, accessibility, active transportation, public space, mobility, equity, active design

An Erratum on Enhancing shared street accessibility in heritage sites for individuals with visual disabilities: a Canadian perspective By Lakoud M, Morales E, Ruiz-Rodrigo A, Feillou I, Mathieu S and Routhier F (2024). Front Rehabil Sci. 5:1419446. doi: 10.3389/fresc.2024.1419446

Due to a production error, the wrong affiliation was listed for reviewer Carla Grião. The corrected reviewer details appear below:

“Carla Grião, Universidade Lusófona, Portugal, in collaboration with reviewer CW".

The publisher apologizes for this mistake.

The original version of this article has been updated.

